# PEI-Mediated Transient Transfection of High Five Cells at Bioreactor Scale for HIV-1 VLP Production

**DOI:** 10.3390/nano10081580

**Published:** 2020-08-12

**Authors:** Eduard Puente-Massaguer, Florian Strobl, Reingard Grabherr, Gerald Striedner, Martí Lecina, Francesc Gòdia

**Affiliations:** 1 Departament d’Enginyeria Química, Biològica i Ambiental, Universitat Autònoma de Barcelona, 08193 Barcelona, Spain; francesc.godia@uab.cat; 2 Austrian Centre of Industrial Biotechnology (acib GmbH), 1010 Vienna, Austria; florian.strobl@boku.ac.at; 3 Department of Biotechnology, University of Natural Resources and Life Sciences, 1190 Vienna, Austria; reingard.grabherr@boku.ac.at (R.G.); gerald.striedner@boku.ac.at (G.S.); 4 IQS School of Engineering, Universitat Ramón Llull, 08017 Barcelona, Spain; marti.lecina@iqs.url.edu

**Keywords:** High Five cells, transient gene expression, polyethylenimine, virus-like particle, bioreactor

## Abstract

High Five cells are an excellent host for the production of virus-like particles (VLPs) with the baculovirus expression vector system (BEVS). However, the concurrent production of high titers of baculovirus hinder the purification of these nanoparticles due to similarities in their physicochemical properties. In this study, first a transient gene expression (TGE) method based on the transfection reagent polyethylenimine (PEI) is optimized for the production of HIV-1 VLPs at shake flask level. Furthermore, VLP production by TGE in High Five cells is successfully demonstrated at bioreactor scale, resulting in a higher maximum viable cell concentration (5.1 × 10^6^ cell/mL), the same transfection efficiency and a 1.8-fold increase in Gag-eGFP VLP production compared to shake flasks. Metabolism analysis of High Five cells indicates a reduction in the consumption of the main metabolites with respect to non-transfected cell cultures, and an increase in the uptake rate of several amino acids when asparagine is depleted. Quality assessment by nanoparticle tracking analysis and flow virometry of the VLPs produced shows an average size of 100–200 nm, in agreement with immature HIV-1 viruses reported in the literature. Overall, this work demonstrates that the High Five/TGE system is a suitable approach for the production of VLP-based vaccine candidates and other recombinant proteins.

## 1. Introduction

Insect cell lines are a well-established platform for the production of a wide variety of recombinant products, including antibodies [1], enzymes [2], hormones [3] and more complex biologicals such as different types of nanoparticles [4,5]. The production strategy typically consists of infecting insect cells with a modified baculovirus (BV) encoding for the gene of interest (GOI). The insect cell/baculovirus expression vector system (BEVS) has proven to be very useful for the production of virus-like particles (VLPs), generally achieving higher nanoparticle yields in comparison to mammalian cell lines [6]. VLPs mimic a virus structure but do not harbor genetic material of the wild-type virus, being exclusively formed by the structured and repetitive self-assembly of one or more virus-derived proteins [7]. Enveloped VLPs are a subclass of these nanoparticles that offer the possibility to display different types of epitopes in their lipid membrane, making them very attractive in cancer immunotherapy [8] and vaccine development [9]. Among them, Gag-based VLPs have received special attention since they can be produced at high levels with the insect cell/BEVS [10]. Nevertheless, several limitations are associated with this system and are principally related to the lytic nature of the BV infection. Disadvantages comprise the early appearance of cell death and consequent release of host-derived proteases, and the need to amplify, maintain and titrate the BV working stock. As for Gag VLPs, the co-production of BV particles that share similar physicochemical properties with VLPs hinders the purification of these nanoparticles. Despite recent advancements have been made in this direction [11,12], difficulties are still encountered to achieve a complete separation between specimens.

Plasmid DNA-based transient gene expression (TGE) has become a powerful alternative to the BEVS given that moderate to high VLP titers can be obtained in a short time frame [13]. TGE consists of the introduction of foreign DNA encoding for a GOI into cells, which is usually achieved by means of positively charged transfection reagents. Once the DNA is introduced, it remains as an episomal element inside cells unless selection pressure, typically an antibiotic, is added to the culture [14]. Therefore, the expression of the GOI is lost over time after cell division. In recent years, several studies have shown that suspension-adapted High Five and Sf9 cells are ideal hosts for the production of reporter proteins [15,16,17], antibodies [18,19,20] and surface proteins [21] in this BV-free environment. Still, the assessment of the insect cell/TGE system to produce more complex products such as VLPs remains to be investigated. 

Polyethylenimine (PEI) has gained progressive relevance as transfection carrier for insect cell/TGE approaches, since transfection efficiencies are high, it is cheaper than the majority of commercial reagents and the overall cost of the bioprocess is reduced [22]. This is of great importance for the production at larger scales in order to meet the increasing demand of therapeutic and diagnostic products. Despite the recent advancements reported for this system, most of the studies dealing with TGE scale-up have been conducted in mammalian cell lines, and there is little information about PEI-mediated insect cell/TGE at this level. Current knowledge about recombinant protein production in insect cells at bioreactor scale is related to the BEVS, with results reported in stirred tank [4,23,24] and wave bioreactors [25,26], and high-volume shake flasks [27]. Therefore, considering the advances reported for TGE in insect cells at small scale, there is a need to evaluate the feasibility of this system at bioreactor scale.

In this work, PEI-mediated TGE of High Five cells is evaluated as a strategy to produce several recombinant products with different complexities, including the intracellular enhanced green fluorescent protein (eGFP) [16], the human secreted alkaline phosphatase (hSEAP) and human immunodeficiency virus type 1 (HIV-1) Gag VLPs. Toward facilitating bioprocess characterization and discriminating VLPs from other nanoparticles, the Gag-eGFP fusion protein is used. VLP production is successfully achieved in a 0.5 L stirred-tank bioreactor, with a detailed study of the metabolism of transfected High Five cells. In an attempt to gain insight into the quantity and quality of the nanoparticles produced, flow virometry and nanoparticle tracking analysis are applied to monitor the High Five/TGE system. 

## 2. Materials and Methods 

### 2.1. Cell Culture Conditions

The suspension-adapted *Trichoplusia ni* BTI-TN-5B1-4 cell line (High Five, cat. num. B85502, Thermo Fisher Scientific, Grand Island, NY, USA) was grown in the low-hydrolysate animal origin-free Sf900III medium (Thermo Fisher Scientific). Cells were subcultured three times a week at a density of 2–4 × 10^5^ cells/mL in 125 mL disposable polycarbonate Erlenmeyer flasks (Corning, Steuben, NY, USA), as previously described [16]. All cultures were grown in an orbital shaker at 130 rpm (Stuart, Stone, UK) and maintained at 27 °C. Cell count and viability were measured with the Nucleocounter NC-3000 (Chemometec, Allerød, Denmark) using acridine orange for cell detection and 4′,6-diamidino-2-phenylindole (DAPI) (Chemometec) to quantify non-viable cells. 

### 2.2. Construction of Plasmid DNA

The plasmid vector used in this work was pIZTV5 (cat. num. V801001, Thermo Fisher Scientific), which harbors the immediate–early *OP*IE2 promoter. The genes encoding for the intracellular enhanced green fluorescent protein (eGFP), the truncated form of the human placental secreted alkaline phosphatase (hSEAP) and the HIV-1 Gag fused in frame to the eGFP were cloned into this vector using standard cloning procedures. Briefly, the hSEAP gene was amplified by PCR from the pUNO1-hSEAP plasmid (Invivogen, San Diego, CA, USA) with the following specific primers: fwd 5′-CGTAGGTACCTCATGATTCTGGGGCCCTGC-3′, rev 5′-CGTAGCGGCCGCGTCCAAACTCATCAATGTATC-3′. The amplified fragment was digested with *Kpn*I and *Not*I and ligated, resulting in the pIZTV5-hSEAP. The Gag-eGFP gene was obtained by digesting the pGag-eGFP plasmid (NIH AIDS Reagent Program, cat. num. 11468) [28] with *Kpn*I and *Not*I obtaining the pIZTV5-Gag-eGFP plasmid after ligation. The pIZTV5-eGFP plasmid was developed as previously described [16]. Plasmid DNA concentration was measured using a Nanodrop 1000 spectrophotometer (Thermo Fisher Scientific). 

### 2.3. Transient Gene Expression in Erlenmeyer Flask

High Five cells were transiently transfected with different DNA plasmids using 25 kDa linear polyethylenimine (PEI, PolySciences, Warrington, PA, USA) according to an optimized protocol reported in a previous work [16]. Briefly, exponentially growing cells were centrifuged at 300× *g* for 5 min and resuspended to 1.5 × 10^6^ cell/mL in 15 mL of pre-warmed Sf900III medium. DNA and PEI polyplex formation was performed in 150 mM NaCl at a final volume of 1 mL with DNA at 2.1 µg/mL added first and vortexed for 10 s. Afterwards, PEI at 9.3 µg/mL (DNA:PEI mass ratio of 1:4.4) was added to DNA, vortexed for 3 s three times and added to the cell culture.

### 2.4. Transient Gene Expression in Bioreactor 

A 2 L DASGIP^®^ Bioblock glass bioreactor (Eppendorf, Hamburg, Germany) equipped with three Rushton impellers was used for High Five cell cultivation in 0.5 L working volume. Aeration was performed through the sparger by air pulses to maintain the dissolved oxygen (DO) at 30% oxygen of air saturation. The air flow rate was set at 1 L/h and temperature at 27 °C. Initial agitation conditions were set at 150 rpm and were automatically adjusted by the DASware control software (Eppendorf) to maintain the DO setpoint at 30% oxygen of air saturation. The pH was fixed at 6.4 and controlled with 20% *w*/*w* H_3_PO_4_ and 7.5% *w/w* NaHCO_3_. Antifoam C (Sigma Aldrich, Saint Louis, MO, USA) was added to the cell culture by pulses to prevent foam formation. 

High Five cells were grown in the incubator to 1 × 10^6^ cell/mL. Prior to inoculation, the medium was exchanged by centrifugation at 300× *g* for 5 min, cells were resuspended in 0.5 L of fresh Sf900III medium and transferred to the bioreactor. Cells were transfected when they reached 1.5 × 10^6^ cell/mL using the standard procedure for DNA:PEI polyplex formation detailed in the previous section. pH control was started the day after transfection in order to avoid interferences with positively charged DNA:PEI polyplexes. 

### 2.5. Flow Cytometry

The percentage of eGFP and Gag-eGFP-expressing cells was assessed using a BD FACS Canto II flow cytometer equipped with a 488 and 635 nm laser configuration (BD Biosciences, San Jose, CA, USA). The number of eGFP and Gag-eGFP positive cells was determined in the FITC-A PMT detector. Briefly, 2 × 10^4^ cells were analyzed per sample at a flow rate of 60 µL/min. Single cells were gated according to side scatter (SSC-H) vs. forward scatter (FSC-A) dot plots and GFP positive cells in comparison to a non-transfected control depending on their mean FITC-A fluorescence intensity. Data acquisition and analysis was performed with the BD FACSDIVA software v.5.0 (BD Biosciences). 

### 2.6. Fluorescence Confocal Microscopy 

eGFP and Gag-eGFP transfected cells were visualized using a TCS SP5 confocal microscope (Leica, Wetzlar, Germany). To do this, cells were stained with 0.1% *v*/*v* of CellMask^TM^ and 0.1% *v*/*v* of Hoechst (Thermo Fisher Scientific) to visualize the lipid membrane and cell nucleus, respectively. A washing step was performed to remove excess dye by centrifugation at 300× *g* for 5 min, and the cells were resuspended in fresh Dulbecco’s phosphate-buffered saline (DPBS, Thermo Fisher Scientific). Samples were placed in 35 mm glass-bottom Petri dishes with a 14 mm microwell (MatTek Corporation, Ashland, MA, USA) for visualization.

### 2.7. HPLC Analyses

Glucose, lactate and phosphate concentrations were measured with an ion-exclusion liquid chromatographic method using a sulfonated polystyrene divinyl benzene column (Aminex HPX-87H, Bio-Rad, Hercules, CA, USA) in an Agilent 1200 series HPLC system (Agilent, Santa Clara, CA, USA). A 0.01 N H_2_SO_4_ solution was used as the mobile phase with a flow rate of 0.45 mL/min [29]. All measurements were performed with an AZURA UV/VIS detector (Knauer, Berlin, Germany) with a refractive index detector temperature of 35 °C. The standard deviation of the technique was determined as 0.31% for glucose, 0.26% for lactate and 1.01% for phosphate measurement. Phosphate uptake rate was calculated taking into consideration the amount of phosphate present in the medium and also the volume of H_3_PO_4_ added for pH control. 

Amino acid concentrations were determined by HPLC after derivatization in a reversed-phase Eclipse Plus C18 column (Agilent) at 40 °C according to manufacturer’s instructions (Agilent). The flow rate was adjusted to 0.64 mL/min and two solvents (solution A and B) were used in the mobile phase. Solution A consisted of 10 mM K_2_HPO_4_ and 10 mM K_2_B_4_O_7_ and solution B of a 45/45/10% *v*/*v*/*v* mix of acetonitrile, methanol and water, respectively [29]. Amino acids were detected at 266/305 nm for fluorenylmethoxycarbonyl derivates and at 450 nm for o*-*phthalaldehyde derivates. The final amino acid concentration was quantified using an internal standard calibration. The standard deviation associated with the measurement of amino acid concentration was 4 ± 1%. 

### 2.8. Analysis of Nanoparticle Production

#### 2.8.1. Nanoparticle Tracking Analysis

Gag-eGFP VLP and total nanoparticle concentration in crude supernatants was measured by nanoparticle tracking analysis (NTA) using a NanoSight NS300 (Malvern Panalytical, Malvern, United Kingdom) equipped with a 488 nm filter module for fluorescent nanoparticle detection. Samples from harvested supernatants at 3000× *g* for 5 min were diluted in 0.22 µm-filtered DPBS and continuously injected into the device chamber through a syringe pump at an average concentration of 10^8^ particles/mL (20–60 particles/frame). Videos of 60 s from independent triplicate measurements were analyzed with the NanoSight NTA 3.2 software (Malvern Panalytical).

#### 2.8.2. Flow Virometry

The Gag-eGFP VLP and total nanoparticle production process was followed by flow cytometry using a CytoFlex LX (Beckman Coulter, Brea, CA, USA) equipped with a 488 nm blue laser for fluorescent particle detection and a 405 nm laser/violet side scatter configuration to improve nanoparticle size resolution. Gating of the different populations was made according to SSC-A vs. FITC-A dot plots and using fresh DPBS and Sf900III medium samples as negative controls. Samples from supernatants harvested at 3000× *g* for 5 min were diluted in 0.22 µm-filtered DPBS and triplicate measurements from independent samples were analyzed with the CytExpert 2.3 software (Beckman Coulter). 

### 2.9. eGFP/Gag-eGFP Measurement by Spectrofluorometry

The supernatants of eGFP and Gag-eGFP transfected cells were sampled once a day by centrifugation at 3000× *g* for 5 min. Pelleted cells were then subjected to three freeze-thaw cycles for intracellular eGFP and Gag-eGFP quantification. Briefly, cell pellets were maintained at −20 °C for 2.5 h, thawed at 37 °C during 0.5 h and vortexed for 5 s three times between cycles. Green fluorescence levels were measured in a Cary Eclipse fluorescence spectrophotometer (Agilent Technologies, Santa Clara, CA, USA) at room temperature as follows: $\lambda_{\mathrm{ex}}$ = 488 nm (5 nm slit), $\lambda_{\mathrm{em}}$ = 500–530 nm (10 nm slit). Relative fluorescence units (R.F.U.) were calculated by subtracting fluorescence unit values of non-transfected cultures. eGFP concentrations were determined using a standard curve developed in a previous study [30]. The equation used to convert R.F.U. to eGFP concentration values is:

eGFP (mg/L) = (R.F.U. − 6.7221)/59.144(1)

where eGFP is the estimated concentration of eGFP protein and R.F.U. is the measured eGFP fluorescence intensity in the samples.

VLP quantification was also performed by an indirect quantification technique [31]:

Gag-eGFP (ng/mL) = (3.254 × R.F.U. − 1.6833) × 36(2)

where Gag-eGFP is the estimated concentration of Gag-eGFP polyprotein and R.F.U. is the measured Gag-eGFP fluorescence intensity in the samples. Conversion of the Gag-eGFP concentration to VLP was performed by assuming that one VLP contains 2500 Gag-eGFP monomers of 87.7 kDa per monomer.

The Sf900III medium and a 0.1 mg/mL quinine sulphate solution were used as control patterns to normalize R.F.U. values between experiments.

### 2.10. hSEAP Quantification

High Five cells transfected with the pIZTV5-hSEAP plasmid were harvested by centrifugation at 3000× *g* for 5 min and cell pellets were disrupted as reported in the previous section. The QUANTI-Blue system (Invivogen), which is based on a colorimetric enzyme reaction, was used to evaluate the alkaline phosphatase activity. To do this, 20 µL of sample were added to 200 µL of pre-warmed QUANTI-Blue solution and incubated at 37 °C for 1 h. The absorbance was measured in a Victor^3^ spectrophotometer (PerkinElmer, Waltham, MA, USA) at a wavelength of 620 nm. Relative activity units (R.A.U.) were calculated by subtracting the absorbance of non-transfected cultures. hSEAP concentrations were determined using a calibration curve based on a linear correlation of known hSEAP (Invivogen) concentrations and the corresponding activity units in R.A.U.:

hSEAP (mg/L) = (R.A.U. + 0.0098)/0.2772(3)

where hSEAP is the estimated concentration of the hSEAP protein and R.A.U. is the measured hSEAP activity units in the samples (Figure S1).

### 2.11. Gag-eGFP Quantification using p24 Enzyme-Linked ImmunoSorbent Assay (ELISA)

The intracellular concentration of Gag-eGFP in transfected High Five cells and in culture supernatants was determined with an HIV-1 p24 ELISA Kit (Sino Biological, Wayne, NJ, USA). Supernatants were harvested by centrifugation at 3000× *g* for 5 min and cell pellets were disrupted as described in the previous section. Samples were incubated in SNCR buffer for 10 min at 70 °C and in 1.5% Triton X-100 for 10 min at 100 °C to disrupt nanoparticles. The substrate solution was prepared by dissolving a SIGMA*FAST* OPD substrate tablet and one urea hydrogen peroxide tablet (Sigma Aldrich) in deionized water at a final concentration of 0.4 mg/mL. An HIV-1 p24 standard of known concentration was also included for Gag-eGFP determination. The reaction was stopped by adding a 625 mM H_2_SO_4_ solution. The absorbance was measured at 492 nm with a reference wavelength at 630 nm in a Tecan Infinite 200 Pro reader (Tecan, Männedorf, Switzerland) [32]. p24 concentration values were corrected according to the Gag-eGFP molecular weight.

### 2.12. Analytical Ultracentrifugation

The supernatant of Gag-eGFP transfected High Five cells at 72 hpt was sublayered with 5 mL of 25% and 8 mL of 45% (*w*/*v*) sucrose (Sigma) solution prepared in DPBS or Dulbecco’s modified eagle medium (DMEM, Thermo Fisher Scientific), respectively. An amount of 10 mL of supernatant was loaded in ultracentrifuge tubes (Beckman Coulter), filled to the top with sterile DPBS, and centrifuged at 4 °C for 2.5 h in a Beckman Optima L100XP equipped with a SW-32Ti rotor set at 31,000 rpm. Samples were taken from each ultracentrifugation fraction and pellets were resuspended in 100 µL of sterile DPBS at 4 °C overnight. All samples were maintained at 4 °C until analysis.

### 2.13. Statistical Analyses

Multiple comparative analyses between different conditions and the control were conducted with the Dunnett’s method. The unpaired Student’s *t*-test was used to compare two separate independent samples. Nanoparticle quantification values from triplicate experiments represent the mean and standard deviations of the average of individual analyses. All statistical analyses were performed with SigmaPlot v.12.0 (Systat Software, San Jose, CA, USA). 

## 3. Results and Discussion

### 3.1. Production of Different Recombinant Products

**Figure 1:**
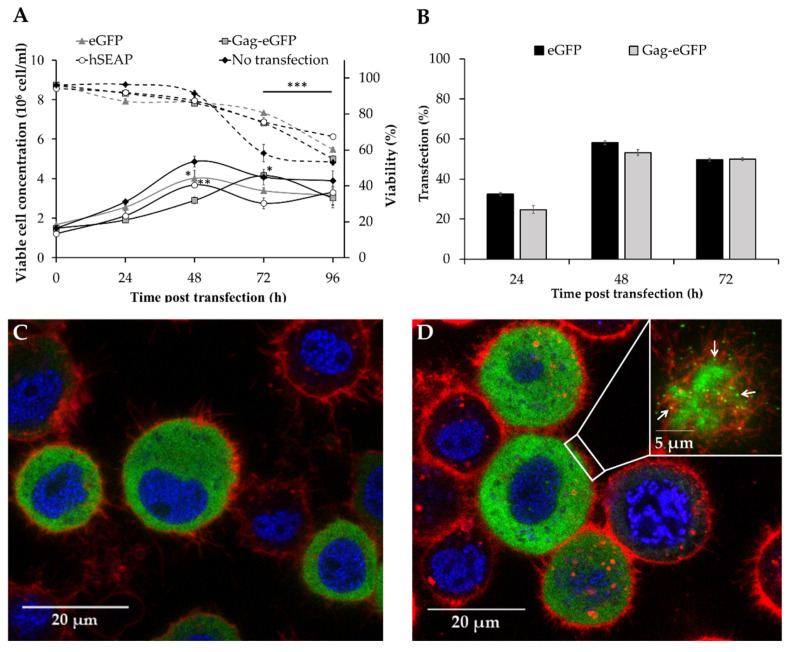
Transient gene expression of eGFP, hSEAP and HIV-1 Gag-eGFP VLPs in High Five cells cultured in shake flasks. (**A**) Cell growth (solid lines) and viability (dashed lines) profiles. (**B**) Transfection efficiencies measured by flow cytometry. (**C**–**D**) Fluorescence microscopy images of transfected High Five cells producing eGFP (**C**) and Gag-eGFP VLPs (**D**). Cell membranes were stained in red with CellMask^TM^ and cell nucleus in blue with Hoechst 33342. VLPs can be observed as green dots (white arrows) budding from cells. Cell nucleus was stained with Hoechst 33342 (blue) and membrane was stained with CellMask^TM^ (red). Mean values ± standard deviation of triplicate experiments are represented. A Dunnett’s test analysis was used to compare the peak of viable cell concentration of the different conditions with the control (no transfection), while a Student *t*-test was performed to evaluate the drop in cell viability between 72 and 96 hpt. * *p*-value < 0.05, ** *p*-value < 0.01, *** *p*-value < 0.001.

The use of High Five cells as a platform to produce simple intracellular recombinant proteins by polyethylenimine (PEI)-mediated transient gene expression (TGE) has been previously demonstrated [16]. The objective in this work is to widen the applicability of the High Five/TGE system for the production of more complex recombinant products, including secreted proteins and multimeric nanoparticles. For this purpose, human-secreted alkaline phosphatase (hSEAP) and HIV-1 Gag-eGFP virus-like particles (VLPs) were selected and compared to the production of intracellular enhanced green fluorescent protein (eGFP). Upon transfection, maximum viable cell concentration was reduced in all cases when compared to the non-transfected condition (Figure 1A), which is probably related to the overexpression of a heterologous product as previously reported for transfected Sf9 cells [30]. The complexity associated with the production of VLPs could be causing the pronounced deceleration of cell growth observed in that case, with cells peaking at 72 hpt instead of the 48 hpt, as observed for the rest of products. In these conditions, a maximum transfection yield of 50–60% was measured for pIZTV5-Gag-eGFP and pIZTV5-eGFP transfected cells at 48 hpt (Figure 1B). Confocal microscopy analysis of pIZTV5-eGFP transfected cells showed that eGFP was intracellularly retained (Figure 1C), while fluorescent nanoparticles (VLPs) could be visualized as green dots (white arrows) in the membrane of pIZTV5-Gag-eGFP transfected cells (Figure 1D, upper right). The latter indicated that transfected High Five cells are capable of correctly processing Gag-eGFP in the form of VLPs, as observed in baculovirus infected insect cells [33,34] and mammalian cell lines [35,36]. 

**Figure 2:**
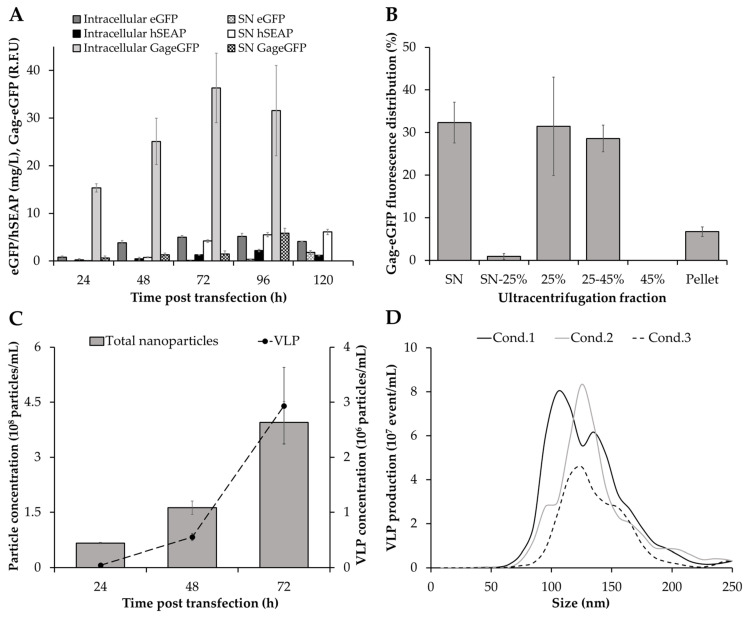
Recombinant protein production in transfected High Five cells cultured in shake flasks. (**A**) Intra- and extracellular production of eGFP, hSEAP and Gag-eGFP. (**B**) Fluorescence distribution of Gag-eGFP by spectrofluorometry in supernatants harvested at 72 hpt after double sucrose cushion ultracentrifugation. (**C**) Analysis of the nanoparticle production process by flow virometry. (**D**) Assessment of VLP size distribution by nanoparticle tracking analysis at 72 hpt. The average values of triplicate experiments are represented.

Maximum eGFP and hSEAP production was achieved at 72 hpt, with the majority of the eGFP produced intracellularly (5.0 ± 0.4 mg/L) and hSEAP secreted to the supernatant (4.2 ± 0.3 mg/L), as expected (Figure 2A). In the same line, the production of Gag-eGFP continuously increased, attaining its maximum concentration at 72 hpt. Notably, analysis of intracellular Gag-eGFP content by spectrofluorometry revealed that a significant amount of the Gag-eGFP produced remained inside the cells and was not being released to the supernatant, thus highlighting the inherent complexity in processing these nanoparticles. Similar results have been recently reported in Sf9 [30] and HEK 293 cells [37], showing a potential bottleneck in processing all the Gag polyprotein produced into VLPs. This evidence possibly indicates that the limiting step in producing these nanoparticles is not cell line but rather product-dependent. Despite Gag-eGFP concentration achieved a plateau at 72 hpt, a 4-fold increase in Gag-eGFP production was measured in the supernatant at 96 over 72 hpt. A significant drop in cell viability was measured in this period, which could explain the increase in Gag-eGFP fluorescence in the supernatant due to leakage from dead cells (Figure 1A). Therefore, the time of harvest was defined as 72 hpt in order to maintain a cell viability at harvest >80% and minimize the amount of non-assembled Gag-eGFP monomer released to the supernatant. In these conditions, the quantity of Gag-eGFP secreted to the supernatant assembled as VLPs accounted for the 60% (Figure 2B). The Gag-eGFP VLP assembly was in the range of that reported for HEK 293 cells by TGE [31] and 4.5-fold higher in comparison to Gag-eGFP VLP production by baculovirus infection in High Five cells [34]. 

Assessment of the VLP production process by flow virometry was in agreement with spectrofluorometry results during the 0–72 hpt period (Figure 2C). VLP production increased up to 72 hpt, attaining a maximum concentration of 2.9 ± 0.7 × 10^6^ VLP/mL (Table 1). Interestingly, a higher VLP yield of 3.6 ± 1.0 × 10^8^ VLP/mL was quantified by nanoparticle tracking analysis (NTA) at the same time, a 2-fold increase in VLP production in comparison to stable Gag VLP producing High Five cell lines [38]. Despite that higher VLP titers were achieved with the baculovirus expression vector system (BEVS) [34], the possibility of producing these nanoparticles in a BV-free environment significantly simplifies the downstream processing, which represents an interesting asset for VLP production. 

The difference in terms of VLP quantification between NTA and flow virometry has also been reported previously [33]. Several studies indicate that the lower nanoparticle levels measured by flow virometry could be the consequence of detecting several nanoparticles as a single larger particle, a phenomenon known as swarm effect [39]. However, it is not clear whether these differences can be fully attributed to this event or to the non-detection of nanoparticles that are below the flow cytometer detection threshold [40].

The presence of extracellular vesicles (EVs) was also observed in supernatants (Figure 2C), confirming that these nanoparticles are concurrently produced with VLPs in High Five cells by TGE. EVs were recently observed in VLP production studies with the BEVS in insect cells [33,34], showing that they are not an exclusive matter of mammalian cell lines [41,42]. Analysis of the average VLP size by NTA resulted in 157.2 ± 8.5 nm (Figure 2D), in agreement with Gag-eGFP VLPs produced in insect cells with the BEVS [43]. EVs displayed a similar mean size of 152.4 ± 15.9 nm than VLPs (*p*-value > 0.05), which raises the need to develop methodologies enabling their separation. Despite recent advancements have been reported by means of chromatographic methods [32,44], difficulties are still encountered in achieving a complete separation between both nanoparticle populations. Furthermore, additional research is required to understand their role and impact in insect cell-based bioprocesses. 

### 3.2. Transferability of VLP Production to Bioreactor

**Figure 3:**
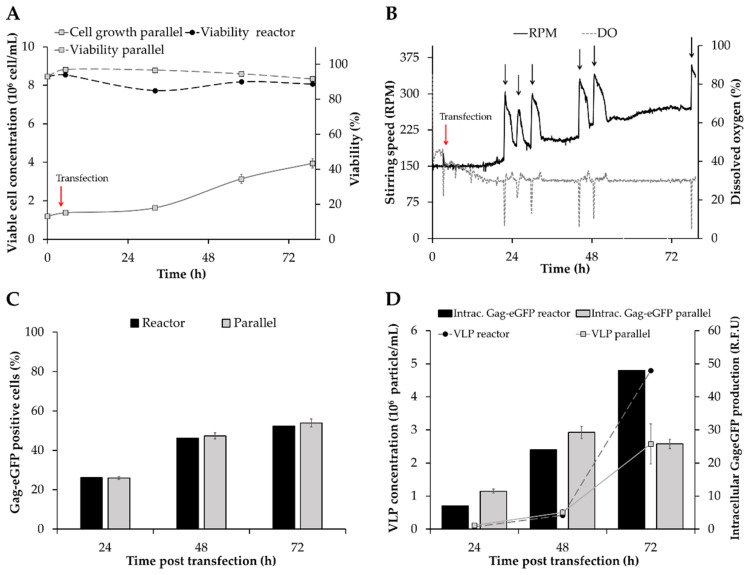
Comparison of Gag-eGFP VLP production in a 2 L DASGIP^®^ Bioblock glass bioreactor and 125 mL Erlenmeyer flasks (parallel). (**A**) Cell growth and viability profile of transfected cultures. The red arrow indicates the time of transfection. (**B**) Evolution of dissolved oxygen and stirring speed requirements of transfected High Five cells. Black arrows show the addition of Antifoam C. (**C**) Percentage of Gag-eGFP positive cells at different time points. (**D**) Analysis of VLP production and intracellular Gag-eGFP content by flow virometry and spectrofluorometry, respectively. Mean values ± standard deviation of triplicate experiments are represented.

A relevant issue in a new bioprocess is the capacity to translate the results to a bigger scale. In this sense, it is essential to prove that the optimal conditions achieved in Erlenmeyer flasks are reproduced at larger scale in a bioreactor. This is highly important for meeting the demands of large amounts of recombinant product for structural or functional studies and pre-clinical testing [45]. High Five cells were inoculated at 1 × 10^6^ cell/mL after medium replacement and transfected with the Gag-eGFP encoding DNA plasmid for VLP production when the viable cell concentration reached 1.5 × 10^6^ cell/mL [16]. In parallel, the same pre-culture was also used in shake flasks as a positive control. No differences were observed in High Five cell growth between bioreactor and shake flask conditions until 48 hpt (Figure 3A). From this point until the end of transfection, cells cultured in the bioreactor attained 5.1 × 10^6^ cell/mL while the shake flask condition achieved a maximum viable cell concentration of 3.9 × 10^6^ cell/mL. These differences in final viable cell concentration could be due to the uncontrolled pH and aeration conditions in shake flasks, resulting in a more unfavorable environment for cell growth [46]. A slight drop in cell viability was measured in the bioreactor at 24 hpt, possibly suggesting that the toxic effect of PEI increased in these conditions. Indeed, shear stress at bioreactor scale can induce a certain degree of cell membrane damage [47], and this could make cultured cells in the bioreactor more susceptible to the toxic effect of PEI. However, cell viability was maintained at >80% in all cases, indicating that High Five cells successfully adapted to the additional stress caused by stirring. Moreover, no deleterious effect on cell viability was observed due to the increasing stirring speeds to maintain the DO level at 30% oxygen of air saturation, highlighting the robustness of this cell line for recombinant protein production in stirred-tank bioreactors. 

High Five cell culture in suspension conditions often requires the addition of anti-clumping agents to decrease the formation of cell aggregates that could impact recombinant product expression [48]. In this study, cell culture in Sf900III medium without the addition of anti-clumping agents resulted in a low level of aggregation, which became more evident in shake flasks at the end of the production phase. As for the bioreactor, no cell clumping was observed, but antifoam addition by pulses was periodically required to prevent foam formation and oxygen limitation (Figure 3B, black arrow). 

Analysis of transfected cells by flow cytometry was conducted every 24 h and resulted in similar transfection efficiencies between both cultivation strategies (Figure 3C). In terms of production, higher concentrations of Gag-eGFP VLPs were quantified by flow virometry in the bioreactor (4.8 × 10^6^ VLP/mL) in comparison to the shake flask condition (2.6 ± 0.6 × 10^6^ VLP/mL) at harvest (Figure 3D). Calculation of the specific productivity in each system yielded a 1.5-fold improvement in VLP (6 × 10^6^ VLP/10^6^ transfected cell·day) but also in intracellular Gag-eGFP production in the bioreactor. This indicates that the larger amount of VLPs achieved in the bioreactor is not only a consequence of a higher viable cell concentration, but the culture conditions are better suited to produce these nanoparticles. These results are in agreement with the VLP productivity increase observed in HEK 293 cells when cultured in bioreactor [49]. An increase of 1.7-fold in VLP production by baculovirus infection of *Tnms*42 insect cells in bioreactor culture conditions has also been reported [50].

Eventually, the quality of VLPs produced in the bioreactor was evaluated by NTA at 72 hpt. In this context, an average VLP size of 163.1 ± 12.7 nm was measured, which is in the range of that observed for shake flask-produced VLPs (*p*-value > 0.05). Likewise, the concomitant production of EVs with a mean size of 160.7 ± 5.8 nm was also detected. 

### 3.3. Analysis of Metabolites

**Figure 4:**
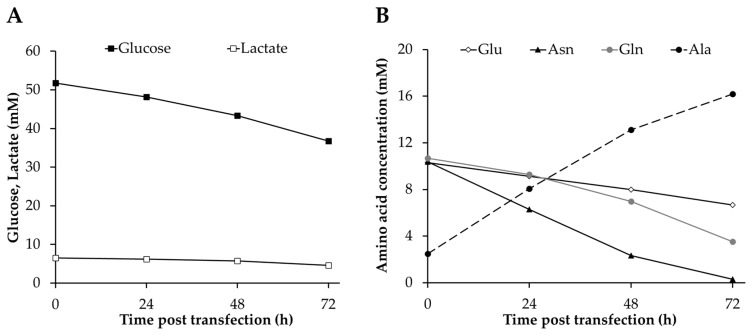
Consumption and production of different metabolites in transfected High Five cells at bioreactor scale. (**A**,**B**) Profiles of glucose and lactate (**A**) and main amino acids produced/consumed (**B**). Glu: glutamic acid; Asn: asparagine; Gln: glutamine; Ala: alanine.

The metabolic profile of High Five cells was analyzed in order to determine the effect of TGE on these cells during VLP production at bioreactor scale and to compare it to parental cells under the same culture conditions. Glucose and glutamine were consumed at high rates (Figure 4), with glucose being preferred over glutamine by 2- to 3-fold in the TGE condition (Table 2), and by 3- to 5-fold in the non-transfected culture (Table S1). None of them was completely exhausted during the bioreactor culture, but the specific glucose consumption rate decreased by 16 and 53% at the end of TGE and in the non-transfected cell culture, respectively, while a similar glutamine consumption level was maintained throughout the experiment. Both metabolites are important energy sources for animal cells via their incorporation into the Krebs cycle through glucose-derived acetyl-coA and glutamine-derived 2-oxoglutarate. Glucose and glutamine consumption rates are lower than those observed in non-transfected cells (Table S1), as well as compared to data reported for baculovirus infected cells [51] and stable insect cell lines [38] in similar culture conditions. The presence of glucose-containing disaccharides, maltose and sucrose, was also detected in the Sf900III medium, but they were consumed at significantly lower rates compared to glucose (data not shown). The consumption of significant amounts of phosphate was also measured during transfection and could be a consequence of the need for lipid biosynthesis for cell growth and VLP production [52]. 

Asparagine was the amino acid consumed at the highest rate (Table 2) and was completely exhausted by the end of the experiment (Figure 4B). Asparagine was consumed more rapidly in the non-transfected cell culture, probably due to the faster cell growth kinetics of parental High Five cells (Figure 1A). The high level consumption of this amino acid in High Five cells for energy generation via oxaloacetate incorporation into the Krebs cycle is well-known and explains the lower glutamine consumption, but the dependence on this amino acid seems to be more pronounced in transfected High Five cells in comparison to baculovirus-infected cells, which tend to consume higher amounts of glucose [46]. Serine biosynthesis was detected at the beginning of transfection when the main carbon and nitrogen sources were not limiting. Likewise, aspartate was initially synthesized by High Five cells, but started to be metabolized at a late stage of transfection, when asparagine became limiting. Interestingly, this behavior of initial biosynthesis of both amino acids was not observed in baculovirus-infected High Five cells [53]. On the other hand, glutamic acid consumption decreased over time, which could be associated with the reduction in asparagine uptake rate as previously reported [54]. The rest of amino acids were consumed to a lesser extent and in the case of tyrosine, proline and the essential amino acids threonine, valine, isoleucine, leucine and phenylalanine the consumption rate increased at the end of transfection. Despite the lower consumption rates, these amino acids have proven to be fundamental for High Five cell maintenance and growth [52]. Alanine was the main by-product generated during cell culture, since this metabolite acts as a nitrogen acceptor under glucose excess conditions [55]. Interestingly, its production rate decreased in parallel to the reduction of asparagine consumption (Table 2 and Table S1) and the concentrations achieved were higher than those observed for baculovirus-infected High Five cells [53], which could be the consequence of a higher asparagine uptake rate. However, no lactate production was detected which differs from previous studies conducted in High Five cells that report substantial accumulation of this by-product [56]. In fact, lactate consumption was measured albeit maintained at low level until the end of the experiment. Similar results were reported in the bioreactor cultivation of Sf9 cells under no oxygen limitation conditions [57]. In general terms, it is possible to observe that metabolite consumption rates are lower for TGE with respect to parental cells, but a re-direction of the energetic sources occurs by the end of transfection to counterbalance the depletion of asparagine, since an increase in the uptake rate of several amino acids is detected. 

## 4. Conclusions

The versatility of the High Five/TGE system for producing recombinant proteins with different complexities is proven in this study. For the first time, the successful production of VLPs using this strategy at bioreactor scale was demonstrated, with no differences in terms of transfection efficiency and a 1.8-fold increase in VLP titer at 72 hpt in comparison to the optimized conditions in shake flasks. The size of Gag-eGFP VLPs obtained corresponds to that observed in VLPs produced with the reference system based on the BEVS. In all cases, the co-expression of EVs with similar sizes to VLPs is observed, which underscores the need to develop efficient separation strategies. Metabolic analysis of transfected High Five cells shows a reduction in the consumption of the principal energy sources in comparison to parental cells and an increase in the uptake rate of several amino acids when asparagine becomes limiting. All in all, the High Five/TGE system provides a valuable approach for accelerating the manufacture of biotechnological products. Moreover, the good performance of this system at bioreactor scale opens the possibility of extending the production phase and increasing the final product yields through the tailored design of perfusion cultivation and re-transfection strategies.

## Figures and Tables

**Table 1:** Gag-eGFP production in shake flasks at 72 hpt using different quantification methodologies.

Quantification Method	Fluorescent Particles/mL	Total Particles/mL	Supernatant	Intracellular
NTA (particles/mL)	3.6 ± 1.0·$10^{8}$	2.4 ± 0.3·$10^{11}$	-	-
Flow virometry (particles/mL)	2.9 ± 0.7·$10^{6}$	4.0 ± 0.6·$10^{8}$	-	-
ELISA (ng/mL)	-	-	17.1	238.4
Fluorometry (R.F.U.)	1.4 ± 0.6·$10^{8}$^a^	-	1.5 ± 0.6	36.3 ± 7.3

^a^ This is the resulting value of correlating R.F.U. to VLP concentration with Equation (2).

**Table 2:** Uptake and production rates of the main metabolites in High Five cells transfected at bioreactor scale for Gag-eGFP VLP production. Rates are expressed in nmol/(10^6^ cell·h) and negative values indicate consumption.

Metabolite	Time Post Transfection (h)		
	0–24	24–48	48–72
Glucose	−90.3	−83.0	−75.6
Lactate	−8.4	−7.8	−13.1
Phosphate	−48.5	−53.2	−17.6
Aspartic acid	25.8	7.1	−5.3
Glutamic acid	−29.0	−19.9	−15.0
Asparagine	−103.8	−68.0	−23.4
Serine	30.0	−0.3	1.8
Glutamine	−35.4	−39.8	−39.8
Histidine	−1.1	−2.2	−2.0
Glycine	−6.2	−7.8	−2.8
Threonine	1.5	−0.8	−2.0
Arginine	−0.7	−5.1	−1.7
Alanine	140.7	86.9	35.3
Tyrosine	0.3	−1.7	−3.0
Valine	0.2	−4.5	−5.7
Methionine	−5.6	−6.2	−5.2
Tryptophan	−0.1	−1.4	−0.9
Phenylalanine	−2.0	−3.7	−3.6
Isoleucine	−1.2	−4.8	−4.4
Leucine	−0.9	−6.1	−5.9
Lysine	−8.0	−17.9	−5.9
Proline	−2.6	−5.1	−4.6

## Supplementary Materials

The following are available online at https://www.mdpi.com/2079-4991/10/8/1580/s1, Figure S1: Linear relation between relative activity units (arbitrary units) and hSEAP concentrations (mg/L), Table S1: Uptake and production rates of the main metabolites in parental High Five cells cultured at bioreactor scale.
