# Supplementary material for: PEI-Mediated Transient Transfection of High Five Cells at Bioreactor Scale for HIV-1 VLP Production
Source: Nanomaterials (Basel). 2020 Aug 12;10(8):1580. doi: 10.3390/nano10081580 (PMC7466501; doi:10.3390/nano10081580)
Supplement: Supplementary file 1 [file nanomaterials-10-01580-s001.pdf]

Article

PEI-Mediated Transient Transfection of High Five Cells at Bioreactor Scale for HIV-1 VLP Production

Eduard Puente-Massaguer, Florian Strobl, Reingard Grabherr, Gerald Striedner, Martí Lecina and Francesc Gòdia

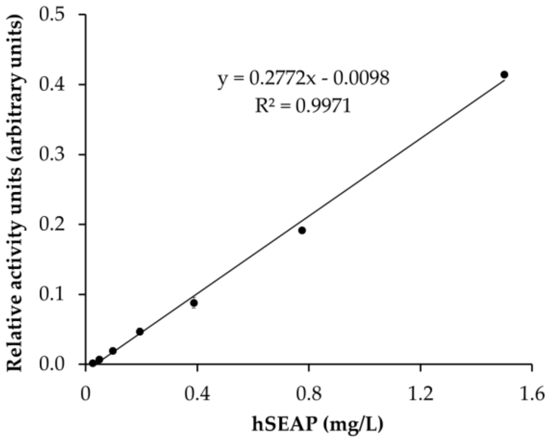

Figure S1. Linear relation between relative activity units (arbitrary units) and hSEAP concentrations (mg/L).

Table S1. Uptake and production rates of the main metabolites in parental High Five cells cultured at bioreactor scale. Cells were seeded at 1 x 10^6 cell/mL and cultured in 0.5 L of bioreactor volume. Rates are expressed in nmol/(10^6 cell·h).

**Metabolite**	**Time (h)**		
	**0 – 24**	**24 – 48**	**48 – 72**
Glucose	-118.5	-95.3	-55.4
Lactate	-17.8	-8.5	0.7
Aspartic acid	14.1	-17.3	-7.1
Glutamic acid	-37.0	-23.1	-7.6
Asparagine	-117.7	-19.7	-
Glutamine	-23.7	-30.0	-19.8
Alanine	161.6	86.9	41.3
